# Intensive In-Bed Sensorimotor Rehabilitation of Early Subacute Stroke Survivors With Severe Hemiplegia Using a Wearable Robot

**DOI:** 10.1109/TNSRE.2021.3121204

**Published:** 2021-11-04

**Authors:** Chunyang Zhang, Mei Zhen Huang, Glenn J. Kehs, Robynne G. Braun, John W. Cole, Li-Qun Zhang

**Affiliations:** Department of Physical Therapy and Rehabilitation Science, University of Maryland, Baltimore, MD 21201 USA; Department of Physical Therapy and Rehabilitation Science, University of Maryland, Baltimore, MD 21201 USA; University of Maryland Rehabilitation & Orthopaedic Institute, Baltimore, MD 21207 USA; Department of Neurology, University of Maryland, Baltimore, MD 21201 USA; University of Maryland Rehabilitation & Orthopaedic Institute, Baltimore, MD 21207 USA; Department of Neurology, University of Maryland, Baltimore, MD 21201 USA; Department of Neurology, Baltimore Veterans Affairs Medical Center and University of Maryland, Baltimore, MD 21201 USA; Department of Physical Therapy and Rehabilitation Science, University of Maryland, Baltimore, MD 21201 USA; Department of Orthopaedics, University of Maryland, Baltimore, MD 21201 USA, and also with the Department of Bioengineering, University of Maryland, College Park, MD 20740 USA

**Keywords:** Stroke, severe hemiplegia, lower extremity, motor recovery, in-bed rehabilitation, ankle robot

## Abstract

Rehabilitation for stroke survivors with severe motor impairment remains challenging. Early motor rehabilitation is critical for improving mobility function post stroke, but it is often delayed due to limited resources in clinical practice. The objectives of this study were to investigate the feasibility and effectiveness of early in-bed sensorimotor rehabilitation on acute stroke survivors with severe hemiplegia using a wearable ankle robot. Eighteen patients (9 in the study group and 9 in the control group) with severe hemiplegia and no active ankle movement were enrolled in acute/subacute phase post stroke. During a typical 3-week hospital stay, patients in the study group received ankle robot-guided in-bed training (50 minutes/session, 5 sessions/week), including motor relearning under real-time visual feedback of re-emerging motor output, strong passive stretching under intelligent control, and game-based active movement training with robotic assistance. Whereas the control group received passive ankle movement in the mid-range of motion and attempted active ankle movement without robotic assistance. After multi-session training, the study group achieved significantly greater improvements in Fugl-Meyer Lower Extremity motor score (p = 0.007), plantarflexor strength (p = 0.009), and active range of motion (p = 0.011) than controls. The study group showed earlier motor recovery for plantarflexion and dorsiflexion than the control group (p < 0.05). This study showed that in-bed sensorimotor rehabilitation guided by awearable ankle robot through combining motor relearning in real-time feedback, strong passive stretching, and active movement training facilitated early motor recovery for stroke survivors with severe hemiplegia in the acute/subacute phase.

## 1. INTRODUCTION

STROKE is a leading cause of long-term disability in the United States [1]. The most common consequence of stroke is motor impairment [2], and motor recovery post stroke is crucial for patients to regain independence [3]. However, rehabilitation for stroke survivors with severe motor impairment remains challenging in clinical practice with substantial demand of resources and limited potential of functional recovery under standard of care [4]. A recent study showed 46.7% patients with severe hemiplegia affecting the lower extremity experienced little or no change in lower extremity motor function during inpatient rehabilitation [5]. To improve functional outcomes and minimize disability post stroke, early intensive rehabilitation plays a critical role [6], [7]. Unfortunately, patients with severe hemiplegia, due to their limited capacity for movement and few options for therapeutic training, are typically left in bed during most of rehabilitation hospital stay [4], [8]. Thus, there is a strong need to develop an in-bed rehabilitation program that can provide intensive sensorimotor therapy to promote motor recovery in severely impaired lower limb poststroke.

Being able to carry out task-specific training with a large number of repetitions and provide objective quantitative assessments, robot-assisted therapy has gained considerable attention in early stroke rehabilitation [9], [10]. However, current lower limb rehabilitation robots are often bulky and require specific patient positioning [11], [12]. Considering that motor recovery is the priority in the early phase of lower limb rehabilitation, there is a strong need to develop a wearable robot suitable for in-bed training, with sensors to detect initial recovering motor signals sensitively, and being able to provide critical real-time feedback to facilitate the motor relearning process.

It has been shown that robotic therapy targeting the ankle joint not only improved mobility function but also promoted better motor recovery in patients post stroke through combined passive-active movement training [13]–[15]. In view of the benefits from robot-assisted ankle training and the need for early rehabilitation, we conducted in-bed sensorimotor training using a wearable ankle robot to facilitate neuroplastic changes and improve motor recovery in stroke survivors with severe hemiplegia, who may otherwise receive little movement therapies under current standard of care. The specially designed ankle robot provides a unique means to detect the presence of early faint motor signals that cannot be discerned by clinical manual evaluations.

This study aimed to investigate the feasibility and effectiveness of in-bed rehabilitation program on stroke survivors with severe hemiplegia and little motor output using a wearable ankle robot. We hypothesized that there would be greater motor recovery for patients receiving robot-guided training (real-time feedback-guided motor relearning training, combined with passive and active movement training) in comparison to controls wearing the same robot who did passive-active movement exercises without robotic guidance.

## II. METHODS

### A. Participants

The study inclusion criteria included: (1) 18–80 years old; (2) first-ever ischemic or hemorrhagic stroke; (3) severe hemiplegia with no visible ankle movement (ankle manual muscle testing [MMT] Grade 0 or 1); (4) within an early subacute phase (< 3 months post stroke). Notably, patients with unstable medical conditions, severe cardiovascular disorders, and severe aphasia who could not follow 2-step instructions were excluded. Participants were assigned into either a study group receiving robot-guided training or a control group wearing the same robot but doing exercises without robotic guidance. The study was approved by the Institutional Review Board of University of Maryland, Baltimore (Protocol HP-00080466, approval date: May 28, 2020). Informed consent was obtained from each participant before the study.

**Fig. 1.**
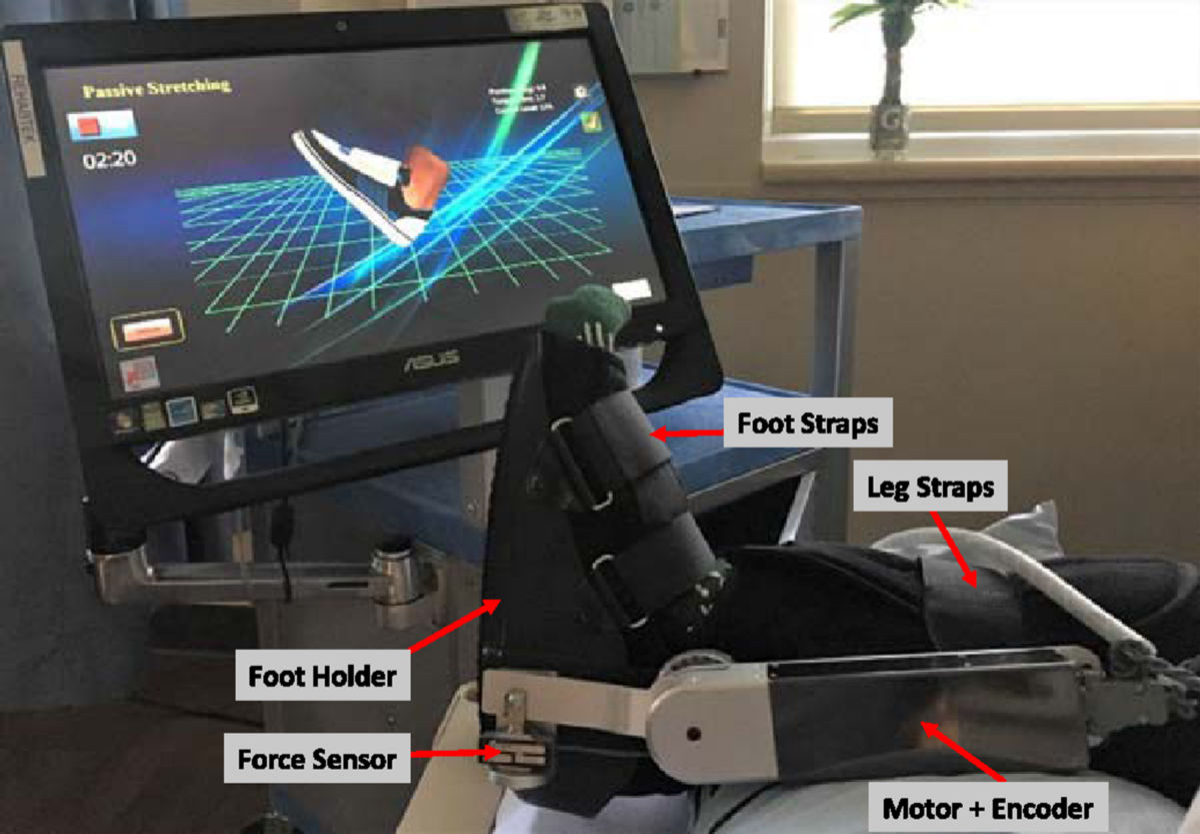
Experimental setup of robot-guided training using a wearable ankle robot and a touchscreen interface. The participant was doing passive stretching under intelligent control. The knee was position at full extension (not shown in the picture).

The wearable ankle robot was taken to the bedside in a cart with a touchscreen computer mounted on a flexible monitor arm. The participant laid on the bed and underwent training in a supine position. Position of the large screen can be adjusted to allow the participant to watch it clearly and comfortably during training. The wearable ankle robot was strapped to the impaired leg with the foot secured to the foot holder and the knee at full extension (Fig. 1). The distal leg of the impaired side was supported by a foam cushion to prevent the heel touching the bed during the ankle movement.

### B. Experimental Setup

**Fig. 2.**
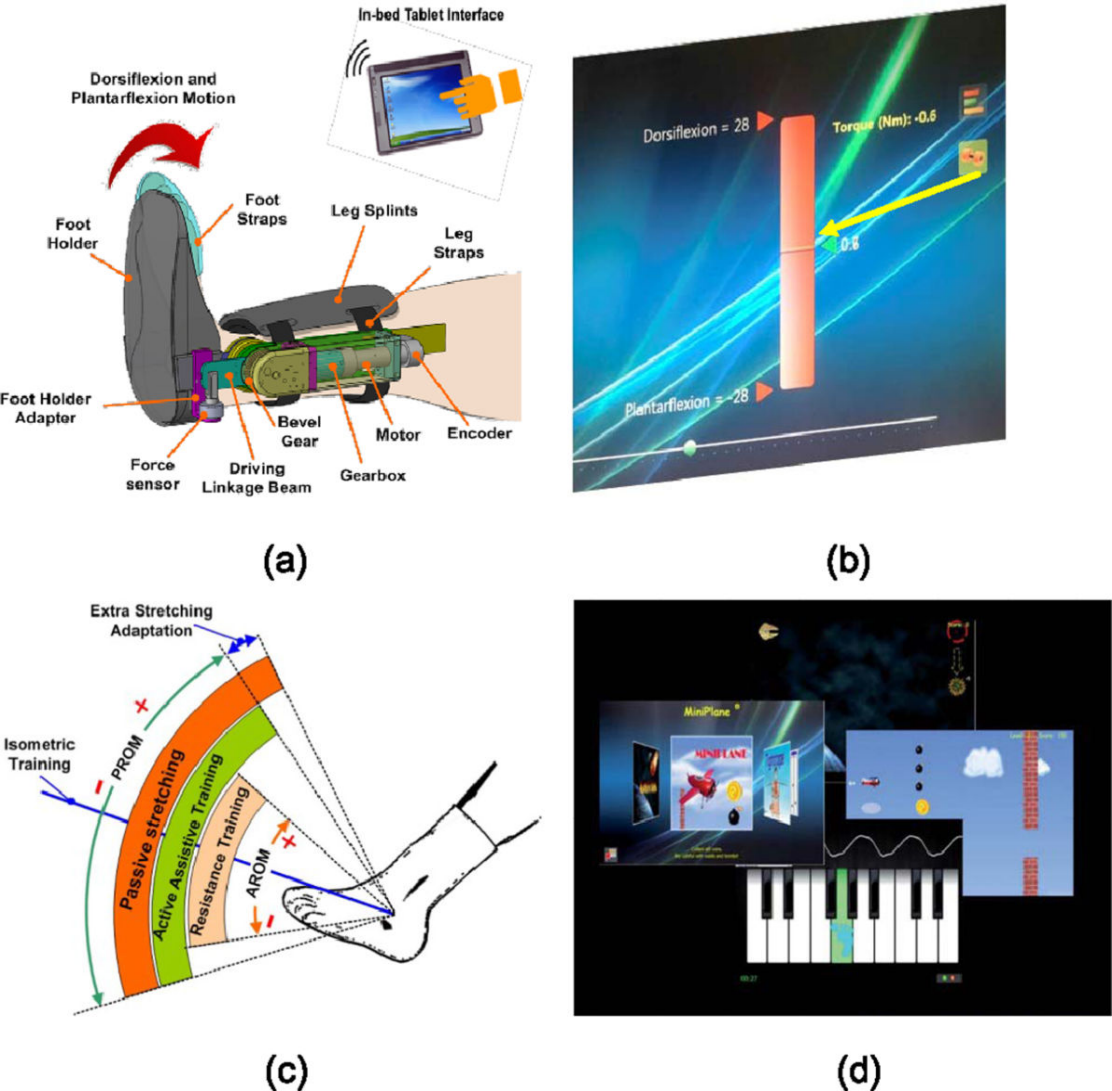
(a) The wearable ankle robot with key components labeled. (b) Real-time feedback of the weak joint torque (indicated by the arrow) with the yellow line becoming a taller bar with increasing torque. (c) Different range of motion involved in the various training modes. Passive stretching was done beyond the passive range of motion (PROM); active movement training with robotic assistance was done in the PROM; active movement training with robotic resistance was done in the active range of motion (AROM); In each mode, the dorsiflexion is defined as “+” direction. (d) Custom designed videogames for active movement training by dorsiflexion or planta flexion of the ankle.

A wearable ankle rehabilitation robot (Rehabtek LLC, Linthicum Heights, MD, USA) was used for in-bed training. The wearable robot was driven by a servomotor (Maxon Powermax, EC-4 poles, 120W) with a gearhead (GP32C, ratio 86:1) and a bevel gear with a ratio of 3:1 (Fig. 2a). The driving linkage was connected to the foot holder through a force sensor (Transducer Techniques, 50 lbs.) to determine the ankle joint torque. The ankle joint movement was measured by an encoder integrated with the motor. The wearable ankle robot featured real-time feedback of ankle joint torque and movement, passive stretching under intelligent control, and robot-guided active movement training with a motivating game interface (Fig. 2). Further technical descriptions can be found in a previous publication [15].

#### 1) Motor Relearning Under Visual Real-Time Feedback:

For patients with no voluntary ankle movement, generating an isometric joint torque was easier than generating ankle movement. We used the wearable robot to lock the patient ankle at an isometric condition and ask the patient to try intentional ankle movement. The isometric joint torque was detected sensitively and fed back to the patient in real time (Fig. 2b). Once an ankle joint torque was generated by the patient, the signal was amplified and displayed on the screen as real-time feedback, which guided the patient to relearn the simple motor tasks after a stroke.

#### 2) Passive Stretching Under Intelligent Control:

The robot was capable to conduct passive stretching under intelligent control. The stretching was driven by a servomotor controlled by a digital signal processor (DSP) [16]. The DSP controller constantly adjusted the stretching velocity *V(t)* based on the measured resistance torque according to Eq. (1), as shown at the bottom of the page. On one hand, in the middle range of motion (ROM) where resistance was low, the motor stretched the relatively slack muscles quickly at higher speeds, for more efficient stretching. On the other hand, the “intelligent stretching” would lower its speed proportionally with increasing resistance torque for safe stretching but would not stop until a preset resistance torque limit was reached. Once the peak resistance torque was reached, the motor would hold the joint at the extreme position for a fixed period of time (e.g., 5 sec) to let stress relaxation occur. The strong stretching and holding were only done at the extreme dorsiflexion position. For safety, position limits were also set and monitored by the DSP controller. However, a small amount of extra stretching movement was used to allow for improvement of the passive ROM beyond the current position limits. The DSP controller adjusted the motor velocity *V(t)* every 0.5 msec according to the following rules, where *θ(t)* and *M*_*res*_*(t)* (with gravitational torque component subtracted) were the ankle position and resistance torque at time *t*, respectively. *M*_*p*_ and *M*_*n*_ were the specified peak resistance torque at the positive and negative ends of the joint ROM, respectively (both were positive numbers). *V*_*min*_ and *V*_*max*_ (two positive numbers) were the magnitudes of the minimum (for stretching in the joint extreme positions) and maximum speed (for stretching in the mid-ROM), respectively. C was a constant, scaling the 1*/M*_*res*_*(t)* to the appropriate stretching velocity. *θ*_*p*_ and *θ*_*n*_ were the specified positive and negative end of the ROM, respectively. *θ*_*d*_ (a non-negative number) represented the allowed further rotation beyond the position limits (to leave room for stretching-induced improvement in ROM). If *θ*_*d*_ was chosen to be a large number (to allow the device move beyond the position limits) or if *θ*_*p*_ and *θ*_*n*_ were set outside the ROM, the stretching control would be dominated by the resistance torque (certainly the stretching would still be safe) and the motor would reverse its rotation once the specific the resisting torque had been reached for the specific amount of time. On the other hand, if *M*_*p*_ and *M*_*n*_ were chosen to be large, the stretching would be restricted by the position limits. In general, we wanted the stretching to reach the torque limits at both ends of the ROM with the position limits incorporated into the control scheme as a safety measure and as an optional mode of stretching; therefore, the *θ*_*p*_ and *θ*_*n*_ would be set to approximately match the ROM by manually pushing the joint to its extreme positions and the *θ*_*d*_ would be chosen as a positive number (e.g., 5°*)*. In this way, the torque limits would be reached most of the time, while the position limits still restrict potential excessive ankle movement. All the control parameters could be changed conveniently within pre-specified ranges.

Overall, the ‘intelligent stretching’ ensured maximal and safe ankle stretching of the impaired ankle. In addition, the strong stretching provided sensory stimulation to help the patient feel the joint and promote motor recovery.

#### 3) Active Movement Training Under Robotic Assistance:

The robot could guide patients to engage in active movement training with custom-designed movement games which were played by patient active ankle dorsiflexion and plantarflexion. The wearable ankle robot was designed to be back-drivable so that patients can move their ankles with little resistance from the device. The ankle robot can detect the movement shortfall by determining the discrepancy between the actual ankle position of the patient and the target position in the game. If the discrepancy exists, the ankle robot would allow the patient try to move the ankle to the desired target position for 2–3 seconds before it provides an assistive torque for the patient to finish the movement task.

Considering that the patients had severe hemiplegia and no active ankle movement, only the assistive training strategy was applied to facilitate motor recovery. If the wearable robot sensed the patient’s attempt or actual movement, it helped the patient finish the movement. If no attempted joint torque was detected, the robot finished the movement after the 2–3 second delay to demonstrate the desired movement. If the patient was able to generate active movement, the robot could also provide resistance to make the training more challenging. The movement assistance and demonstration of the desired movement by the wearable robot played an important role in engaging the severely impaired patients to regain the active movement capacity.


(1) $$V(t)=\{\begin{array}{cc} 0, & \;if\;\left(M_{res}(t)\geq M_{p}or\theta (t)\geq \theta_{p}+\theta_{d}\right)\;and\;need\;to\;hold\;\\ -V_{\max}, & \;if\;\left(M_{res}(t)\geq M_{p}\;or\;\theta (t)\geq \theta_{p}+\theta_{d}\right)\;and\;have\;held\;long\;enough\;\\ \max\left(\frac{C}{M_{res\;}(t)},V_{\min}\right), & \;if\;0<M_{res}(t)<M_{p}\\ \min\left(\frac{C}{M_{res}(t)},-V_{\min}\right), & \;if\;-M_{p}<M_{res}(t)<0\\ V_{\max}, & \;if\;\left(M_{res}(t)\leq -M_{n}\;or\;\theta (t)\leq \theta_{n}-\theta_{d}\right)\;and\;have\;held\;long\;enough\;\\ 0, & \;if\;\left(M_{res}(t)\leq -M_{n}or\theta (t)\leq \theta_{n}-\theta_{d}\right)\;and\;need\;to\;hold\;) \end{array}$$

Interactive games were used to motivate patients to move their ankle in the game plays, and feedback was provided in a video game scenario on a large screen monitor (Fig. 2d). First, there were two kinds of active movement games, one kind was that the robot provided assistance to the desired movement and the other kind was the robot provided resistance to further challenge the patients. Dependent on the patient’s motor ability, assistance or resistance would be provided. Second, the level of movement assistance including the duration of initial no-assistance period (to let the patient try as much as he/she could) and the assisted torque or movement speed after this initial patient trying period was customized based on the patient’s movement ability. For example, patients flew a plane through gaps by moving their impaired ankle up and down. If the patient had difficulty in reaching the target in the game, the robot let the patient try to make the movement for a few seconds, then provided assistance if needed to help the patient reach the target. The patient tried hard to reach the target and might not be aware of the robotic assistance, which was useful to keep the patient engaged in the game-based training. Third, similarly, the level of movement resistance was customized based on the patient’s movement ability and adjusted based on the patient’s progress. Fourth, different movement games including plane flying, piano playing, and tight rope walking (see example games in Fig. 2d) were used to keep the patients engaged, which was important in motor recovery. Fifth, patients also had their preference and selections of the different games. Some like music and thus preferred the Piano game, for example. With the motivating games, the patient was more focused, which was a key in motor recovery training. Patients could also finish with higher number of movement repetitions, which was needed to have longer lasting effect.

#### C. Training Procedures

**Fig. 3.**
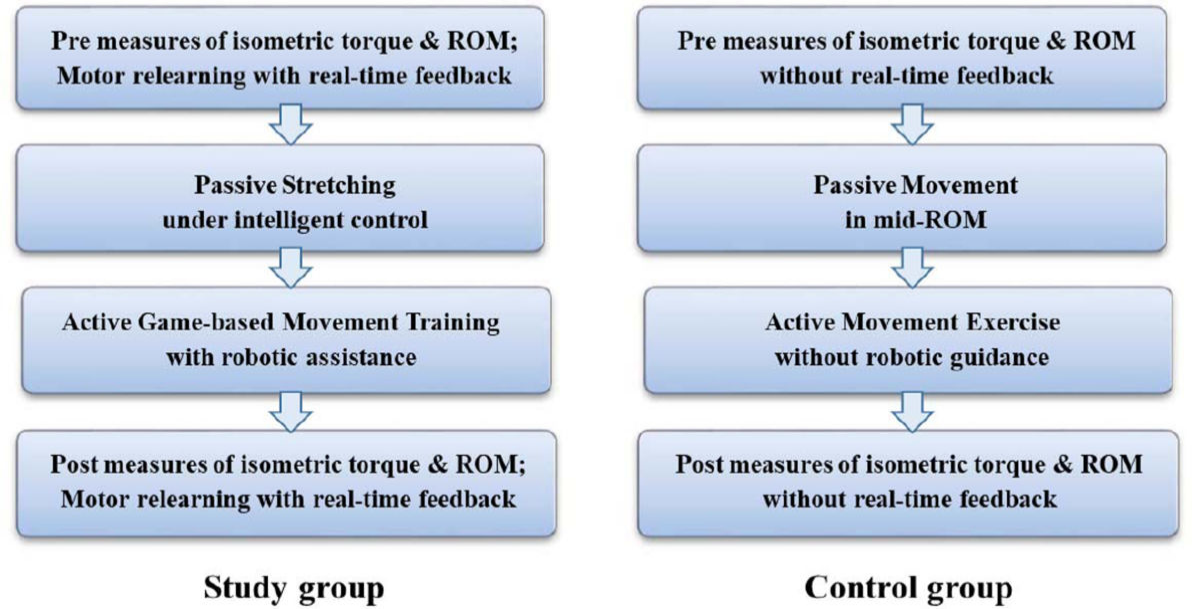
A flowchart of the training protocol for the two groups using the same wearable ankle robot. The signals used in real-time feedback motor relearning include the ankle isometric torque and joint movement.

Participants in both groups were scheduled to participate in hourly training sessions, 5 sessions per week during a typical 3-week stay in the rehabilitation hospital, for a total of about 15 sessions. The training protocol was adjusted individually to accommodate the needs for patients with severe hemiplegia, including more passive stretching (∼20 minutes) and motor relearning training (∼15 minutes), and less active movement training (∼10 minutes), compared with our previous study involving less severe patients [15]. Therapy intensity was maintained ∼150 repetitions for each training session. The training procedures were shown in Fig. 3. All participants received standard of care of inpatient rehabilitation including typical physical therapy and occupational therapy daily sessions.

Participants in the study group received passive stretching vigorously under intelligent control to loosen stiff muscles and provide strong sensory stimulations. With the muscles more compliant and controllable after stretching, the participant was asked to use these muscles immediately to do active game-based movement training with the robot assistance, aiding keep them focused and engaged.

At the beginning and the end of each training session, motor relearning training was conducted by asking the patient to generate isometric ankle joint torque and active range of motion (AROM) evaluations with real-time visual feedback. With the robot held the ankle at an isometric condition, participants were asked to get ready, keep focused and try to plantarflex and dorsiflex the ankle maximally and the isometric joint torque measured sensitively was displayed on a large monitor in real time. This visual feedback was particularly useful to help patients try various ways to regain the ability to generate motor output without observable movements. Similar AROM test was attempted with real-time feedback of the ankle movement.

Participants in the control group received passive ankle movement in the mid-ROM (defined as about half of the ROM measured manually at the beginning of a training session) to reduce the stretching intensity. After the passive movement training, participants in the control group were asked to move their ankle back and forth at their comfortable pace as active movement training. The wearable robot was made free to move through back-drivable control but would not provide any assistance. The participants were instructed to attempt the movement with interval breaks whether they could make the actual ankle movement or not. In general, the amount of passive stretching and active movement training were adjusted based on the patients’ impairment and ability to move for both groups. The ankle muscle strength and AROM were also measured for the control group in each training session but without real-time visual feedback.

#### D. Assessments

Clinical and biomechanical outcome measures were obtained for all the participants before and after the multi-session robot-aided training. Fugl-Meyer Lower Extremity motor score (FMLE) was used to evaluate motor recovery (maximum score of 34) with a higher score indicating better motor recovery [17]. Spasticity of the plantarflexor was evaluated using the Modified Ashworth Scale (MAS) and the scores of 0, 1, 1+, 2, 3 and 4 were adjusted to 0–5 scores [18].

Biomechanical outcome measures were conducted using the wearable robot including the ankle AROM and muscle strength in plantarflexion and dorsiflexion. The AROM was measured by asking the participant to voluntarily move the ankle between the maximal dorsiflexion and maximal plantarflexion. Strength of the dorsiflexors and plantarflexors was measured as maximal voluntary contraction (MVC) by locking the footplate at the 0° dorsiflexion and asking the participant to dorsiflex maximally then plantarflex maximally. Each measure was done 3 times with a rest break of 30–60 seconds to minimize fatigue. The measured data were saved in the robot computer and the averaged value of the 3 assessments was taken as the corresponding outcome measure.

The plantarflexion motor recovery time was determined based on the detection of the plantarflexor motor output during the MVC tasks with real-time joint torque feedback. The dorsiflexion motor recovery time was similarly determined.

#### E. Sample Size Estimation

Our previous study showed that training guided by the same wearable ankle robot induced significant improvements in FMLE, dorsiflexion and plantarflexion MVC and AROM in ankle with effect sizes (Cohen’s d) of 0.52–0.97 (i.e., medium to large effect sizes) [15]. Also, it has been reported that the required sample size significantly decreases with the increase of stroke severity [19]. Thus, we assume a large effect size (convention: f = 0.25) of robotic ankle training for stroke survivors with severe hemiplegia in this study. Based on a 2 × 2 repeated measures analysis of variance (ANOVA), with an alpha value of 5%, power of 80% and attrition rate of 10%, the minimum sample size required to detect a significant group-time interaction effect would be 18 participants (9 participants in each group).

#### F. Statistical Analysis

Although the Shapiro-Wilk test showed that the outcome measures were not normally distributed, F-test suggested it was robust enough that the non-normal distributions were likely to represent reliable data [20]. A 2 × 2 mixed repeated measures ANOVA model, with time (baseline vs. postintervention) as the within-participant factor and group (study vs. control) as the between-participants factor, was used to examine the effects of ankle robot training on the FMLE scores, strength of plantarflexors and dorsiflexors and ankle AROM. Post-hoc pairwise comparisons with Bonferroni corrections were conducted for each group if a significant interaction effect was identified. Baseline characteristics comparisons between the two groups were conducted using t-test or Fisher’s exact test.

The Kaplan-Meier survival analysis with Breslow test was used to compare the recovery time of the plantar- and dorsiflexion motor output between the two groups [21], [22]. Participants who did not show motor recovery were censored at the date of last training session. All statistical analyses were performed using the SPSS statistical software (IBM, Version 26). The statistical significance was set at p<0.05.

## III. RESULTS

Eighteen patients in an early subacute phase post stroke participated in the study with 9 patients in each group. Characteristics of the participants were summarized in Table I. The demographics and baseline outcome measures had no significant difference between the two groups (p>0.05). The in-bed robotic ankle training was well tolerated in this subgroup of severely impaired patients. There were no adverse events related to robotic training. Participants enrolled in this study were all engaged in training until being discharged.

**Fig. 4.**
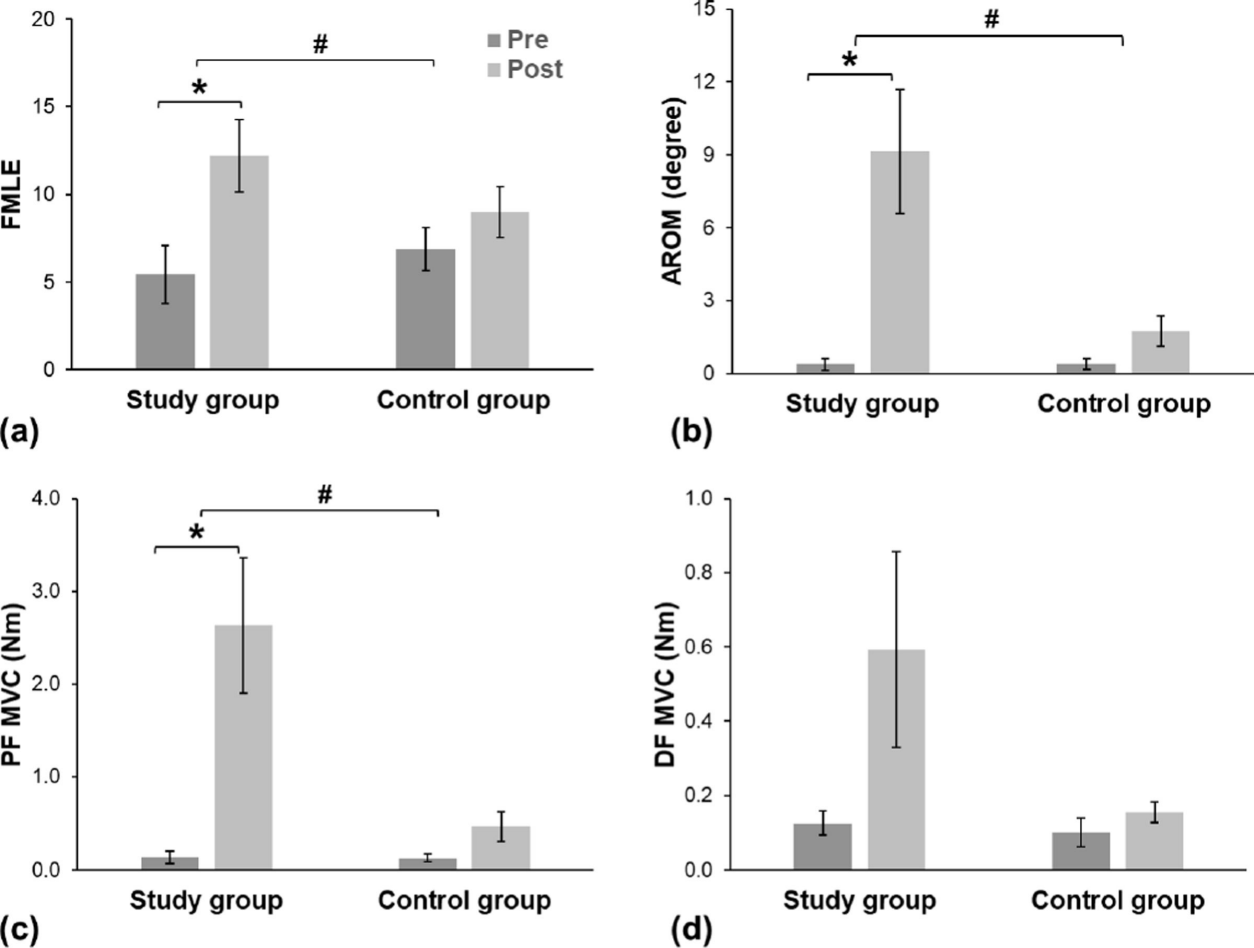
Outcome measures pre- and post-intervention training for the study group and control group. (a) Fugl-Meyer Lower Extremity motor score (FMLE), (b) Active range of motion (AROM) of the impaired ankle, (c) Plantarflexor maximal voluntary contraction (PF MVC), (d) Dorsiflexor maximal voluntary contraction (DF MVC). * denotes significant within-group pre-post difference (p<0.05), while # indicates significant between-group differences (p<0.05). The error bar represents the standard error of the mean.

The 2 × 2 ANOVA revealed significant time × group interaction effects in FMLE (F_1,16_ = 9.653, p = 0.007, *η*2 = 0.376), ankle AROM (F_1,16_ = 8.273, p = 0.011, *η*2 = 0.341) and plantarflexor MVC (F_1,16_ = 8.878, p = 0.009, *η*2 = 0.357), driven by significant increases of FMLE, AROM and plantarflexor MVC in the study group (p<0.01) but not in the control group (p>0.05) (Fig. 4). For dorsiflexor MVC, neither a significant interaction effect nor an overall time effect was observed (Fig. 4). However, the study group showed an average increased torque of 0.46 Nm after training, while the control group had 0.05 Nm increase on average (Table I).

**Fig. 5.**
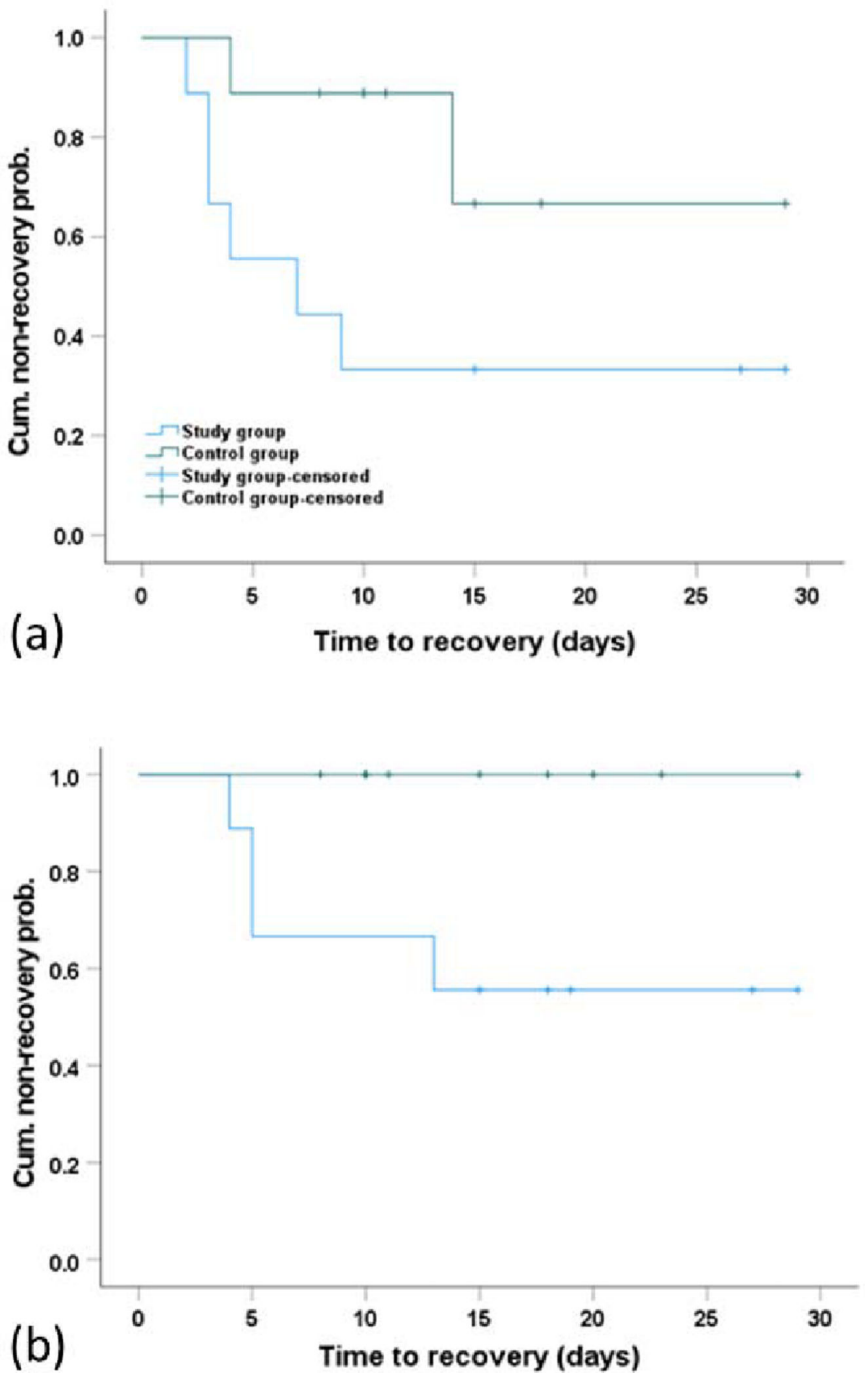
Survival plots of the time to motor recovery using the Kaplan-Meier method with Breslow test. (a) Survival plot of the plantarflexion torque recovery time. The study group showed significantly earlier motor recovery than the control group (p = 0.035). (b): Survival plot of the dorsiflexion torque recovery time. The study group showed significantly earlier motor recovery than the control group (p = 0.041). Cum.: cumulative; prob.: probability.

Six patients in the study group and two in the control group showed plantarflexion torque recovery by the end of training. Using the Kaplan-Meier method with Breslow test, the study group showed significantly earlier motor recovery for plantarflexion torque recovery than the control group (median 7.0 days, 95% confidence interval [CI] 0 – 15.8 days versus not reached; p = 0.035) (Fig. 5a). For the dorsiflexion torque recovery, 4 patients in the study group and none of the control group showed recovery. The study group showed earlier motor recovery for dorsiflexion torque recovery than the control group (p = 0.041) (Fig. 5b).

## IV. DISCUSSION

This study investigated in-bed rehabilitation using a wearable ankle robot for early subacute stroke survivors with severe hemiplegia. To our knowledge, this is the first study demonstrating the feasibility and efficacy of early in-bed robot-guided rehabilitation for lower limb motor recovery in patients with severe hemiplegia and little motor output (MMT = 0 or 1). Our results showed that robot-guided in-bed training was well tolerated in severely impaired patients and could facilitate lower limb motor recovery in the early phase post stroke by combined rehabilitation approaches with real-time visual feedback, controlled passive stretching, and assistive-active movement training. The ankle motor recovery was significantly improved in terms of objectively assessed ankle muscle strength and AROM. Moreover, the significantly increased FMLE motor scores (∼10 points increase) in the study group showed this training could induce lower limb motor recovery in these clinically challenging patients.

The wearable ankle robot with sensitive force and motion sensors implementing an “assist-if-needed” algorithm and visual feedback provided a unique approach for guided motor relearning [15]. For severely impaired patients, once the re-emerging faint and possibly inconsistent motor output signal was detected during the intended voluntary movement, the robot would display the change on the screen and demonstrated the intended motor action to the patients. This visual real-time feedback on torque generation provided useful guidance and motivation to the patients, especially at an early motor re-learning stage [23].

Active physical and cognitive engagement of patients during therapy also play a critical role in motor function recovery [24]. This wearable ankle robot allowed more advanced interaction control with a convenient interface, ranging from passive stretching to active-assisted movement training (up to 100% robotic assistance) for these severely impaired patients [15]. In this study, active movement training may act as motor relearning training, where the participants tried to plan and control their ankle movement. If they were unable to generate the intended motor output, the robot guided them in the attempt and demonstrated the proper movement with up to 100% robotic assistance. If they were able to generate some intended motor output, the robot had the sensitivity to detect the generated torque and provided necessary assistance for the participants to finish the movement.

The forceful stretching under intelligent control is another feature of this wearable ankle robot, which ensured the impaired ankle was stretched throughout its ROM to extreme dorsiflexion and plantarflexion (the true end point) [25], [26]. This not only allowed loosening of stiff ankle muscles, but also delivered a strong sensory stimulation to the impaired leg. As a result, forceful stretching by the robot may enhance sensory input (mainly kinesthetic stimulation), promote sensory integration and motor control in an early phase of motor recovery. Particularly for those with severe hemiplegia, the potential residual neural circuit may route sensory stimulation signals to the brain and help rewire the impaired pathway [7] or reorganize impaired motor cortex to initiate and promote descending motor commands [27]. The passive stretching prior to active movement training helps loosen spastic muscles and prepare them for peripheral motor actions. Centrally, it may produce a priming effect on the impaired motor cortex to facilitate motor output generation [28]. Overall, the combined rehabilitation approaches including passive stretching and active movement seemed to be an efficient strategy to improve early motor recovery [6].

The first months post stroke have been suggested as a critical time window for motor recovery [6]. However, few stroke rehabilitation studies have been conducted during this period [29]. Considering the capacity of robotic rehabilitation, several studies have focused on robot-assisted mobility training in the early phase after an acute stroke [13], [30], [31]. Compared with conventional rehabilitation, these robot-assisted therapies provided early intensive rehabilitation and improved the outcomes of lower limb muscle strength, motor control and gait patterns. However, these well-designed robots may not be a fit for stroke survivors with severe lower limb impairment, due to potential device access difficulty [30], used in a seated position and without strong stretching [13], or required exercise performance ability for multiple joints [31].

In this study, we also observed that the plantarflexion motor reemergence in the study group was significantly earlier than that of the control group. Moreover, plantarflexion motor reemergence seemed to occur earlier and more frequently than that of dorsiflexion motor recovery in both groups. This may be related to the relatively larger volume of plantarflexors than dorsiflexors. Additional interventions aimed at dorsiflexion motor recovery such as the functional electric stimulation of the dorsiflexors may be needed, which may enhance peripheral nerve activities and facilitate motor recovery [10], [32], and help reduce potential foot drop and improve gait [33].

This study has limitations. First, this study had a small sample size and the group assignment was not randomized and blinded. While the present study provided supporting results on patients with severe hemiplegia, a randomized controlled trial with a large sample size and follow-up is needed in the future. Second, the intervention was limited by the length of acute rehabilitation hospital stay, which might be shortened by insurance coverage, patient/family preference, or other factors. Despite the relative short period of the intervention, we did observe significant improvements after robot-guided training. Third, the type of stroke (ischemic or hemorrhage) was not reported due to incomplete information, which may influence the results. Lastly, we only had clinical scales and biomechanical measures in the present study, without neurological imaging measures to investigate neuroplastic changes in the brain. Also, cognitive status changes could influence the motor performance post stroke [34]. Neuro-physiological measures and cognitive evaluations should be included in the future studies.

## V. CONCLUSION

This study showed that early in-bed intervention with a combination of motor-relearning under real-time visual feedback, passive strong stretching, and active movement training guided by the wearable ankle robot was an effective rehabilitation strategy for motor recovery in the acute to early subacute stages post stroke. The intervention is potentially important for patients with severe hemiplegia and little motor output who otherwise have few therapy options.

## Figures and Tables

**TABLE I:** Demographics and Outcome Measures

	Study group (n=9)	Control group (n=9)	*P*
**Gender (men/women)**	7/2	4/5	0.335
**Age (year)**	58.2±12.1	55.6±18.9	0.726
**Affected side (left/right)**	5/4	8/1	0.294
**Time post stroke (day)**	28±19	25±17	0.699
**Training session**	13±3	10±9	0.259
**PF MAS (range:0–5)** ^a^			
Baseline	2 (2–3)	3 (2–3)	0.750
Post-training	3 (2–3)	2(1-2)	0.101
**FMLE (range:0–34)** ^a^			
Baseline	4(2–11)	7 (4–10)	0.492
Post-training	14 (7–18)	9(4–13)	0.007 ^b^
**A ROM of ankle (degree)**			
Baseline	0.38±0.79	0.39±0.69	0.959
Post-training	9.15±7.65	1.75±1.90	0.011^b^
**PF MVC (Nm)**			
Baseline	0.13±0.21	0.13±0.13	0.929
Post-training	2.63±2.18	0.47±0.47	0.009 ^b^
**Dorsiflexor MVC (Nm)**			
Baseline	0.13±0.10	0.10±0.11	0.631
Post-training	0.59±0.79	0.15±0.08	0.124 ^b^

a Median (1st quartile - 3rd quartile range)

b Time-group interaction effects AROM: active range of motion; FMLE: Fugl-Meyer Lower Extremity motor score; MAS: modified Ashworth Scale; MVC: maximal voluntary contraction; PF: plantarflexor
